# COVID-19 Vaccine Acceptance among Ajman Undergraduate Dental Students: A Cross-Sectional Study

**DOI:** 10.1155/2023/3511960

**Published:** 2023-05-29

**Authors:** M. A. Jaber, M. Abdelmagied, E. M. El-Ameen, K. I. Afrashtehfar

**Affiliations:** ^1^Clinical Sciences Department, College of Dentistry, Ajman University, P.O. Box 346, Ajman City, UAE; ^2^College of Medicine, Ajman University, P.O. Box 346, Ajman City, UAE; ^3^Department of Reconstructive Dentistry and Gerodontology, School of Dental Medicine, Faculty of Medicine, University of Bern, Bern 3010, Switzerland; ^4^Artificial Intelligence Research Center (AIRC), Ajman University, P.O. Box 346, Ajman City, UAE

## Abstract

**Background:**

Achieving widespread coronavirus disease 2019 (COVID-19) vaccination is crucial in controlling the pandemic caused by severe acute respiratory syndrome coronavirus 2 (SARS-CoV-2). This cross-sectional study aimed to identify factors associated with the willingness of dental medicine students to receive the COVID-19 vaccine.

**Objectives:**

The study sought to assess the knowledge, attitudes, and behaviors of undergraduate dental students toward COVID-19 vaccines and to identify determinants, motivators, and barriers to vaccine uptake and booster receipt.

**Methods:**

A web-based survey was distributed to all 882 undergraduate dental surgery students in January 2022, and 70.7% of the students responded. The survey used *χ*^2^ tests and logistic regression analysis to examine the association among the variables. The significance level was set at *α* = 0.05.

**Results:**

Most participants (72.4%) reported having adequate knowledge of COVID-19. The vaccine acceptance rate was higher among male and older trainees, with no significant difference compared to women and younger trainees with no significant difference (*p* = 0.849). Acceptance of the vaccine varied according to study level (5-year program), ranging from 44.8% to 73.0%, in the following order 4th > 1st > 3rd > 5th > 2nd year. Social media (76.8%), government websites (66.5%), and family and friends (57.2%) were the main sources of COVID-19-related information. Among hesitant and unwilling participants, the main concerns were side effects (34.0%) and lack of understanding about the vaccine's mechanism (67.3%).

**Conclusions:**

Ajman dental students had moderate knowledge of COVID-19 and obtained information mainly from social media, government websites, and family and friends. Age, sex, and study year influenced vaccine acceptance. The main reasons for refusal were lack of knowledge, fear of side effects, and complications. Education campaigns are needed to increase vaccine acceptance among dental students.

## 1. Introduction

Coronavirus disease 2019 (COVID-19) was officially declared a global pandemic by the World Health Organization (WHO) on March 11, 2020. The magnitude of the pandemic was incomprehensible, and the presence of the COVID-19 virus led to unprecedented challenges in the healthcare system across the globe [1, 2]. The modus operandi to reduce the transmission has been behavioral, such as social distancing, hand sanitization, regular washing of hands, and implementation of personal protective equipment, primarily wearing masks. The COVID-19 virus is more easily transmitted and more highly contagious than its predecessors (the betacoronavirus group), including the Middle East respiratory syndrome coronavirus and severe acute respiratory syndrome-associated coronavirus (SARS-CoV), which remains as a critical difference [3].

COVID-19 infection is characterized by high fever and cough. In contrast, in advanced infectious cases, patients may develop respiratory distress, whereas other reported symptoms include nausea, vomiting, diarrhea, muscle joint pain, and loss of appetite [4–6]. Insufficient comprehension of the COVID-19 process among the general public, healthcare workers, and researchers (such as epidemiologists and immunologists) has endangered timely medical intervention, thereby risking patients' lives [7, 8]. A large amount of information misleading the general public about COVID-19 has spread primarily through social media. Misconceptions and conspiracy theories have globally hampered the COVID-19 understanding among health profession students [9, 10].

The searches for “vaccine” have reached an all-time high globally, and according to the WHO, at least 198 COVID-19 vaccines are undergoing development, with 44 currently being clinically evaluated [11]. A safe and effective anti-COVID-19 vaccine would go a long way toward helping society return to its prepandemic normal. However, despite the recommendations for the general population and the mandate for healthcare professionals, a sector of this population is displaying hesitancy or unwillingness to take the experimental vaccine.

Timeliness of vaccine availability and application reaching herd immunity are not the only challenges from a public health standpoint. Once a vaccine is developed, a sufficient share of the population must be vaccinated to reach significant protection to prevent broader spread in the community. A growing radicalized antivaccination (anti-vax) movement threatens such efforts in North America [12], Europe [13], and Asia [14]. It has been suggested that the community acceptance of the COVID-19 vaccination may not reach the thresholds necessary to achieve herd immunity, estimated at 70% for this particular disease [15]. For instance, a study reported that 49% of the surveyed participants willingly would take a vaccine once available, 31% were hesitant, and 20% would reject being vaccinated (i.e., jabbed) [16]. In another survey, 71% of American adults would voluntarily be vaccinated [17]. Although this type of study helps gauge the potential vaccine acceptance, the surveys lack considering potential attributes that influence public acceptance, such as vaccine efficacy, development, and adverse effects risks.

While COVID-19 vaccination is crucial in controlling the pandemic caused by SARS-CoV-2, there is a knowledge gap in understanding the factors that influence individual preferences about vaccination, particularly among healthcare students such as dental surgery trainees. This study aims to address this knowledge gap by identifying the factors associated with the willingness to receive the COVID-19 vaccine among dental students.

The importance of this study lies in the fact that it can help inform public health policies and campaigns that aim to increase COVID-19 vaccine uptake among dental students and the wider community. By identifying the factors that influence vaccine acceptance among dental medicine trainees, the study can provide insights into the messages and incentives that would be most effective in promoting vaccination. Furthermore, the study's findings could raise awareness among dental students about clinical judgment and risk perception, potentially leading to more informed vaccination decisions. Ultimately, the study's findings could contribute to the global effort to contain the COVID-19 pandemic.

## 2. Material and Methods

### 2.1. Ethical Approval and Registration

This descriptive cross-sectional study started after its study protocol obtained approval from the Review and Ethical Committee of the educational institution (#DHF-2020-04-27) while following the ethical guidelines outlined in the Declaration of Helsinki relating to biomedical research involving human subjects, which sets forth standards for research involving human subjects, including informed consent, confidentiality, and participant welfare. The study was conducted during the first week of January 2022 at Ajman University (AU), UAE. All the voluntarily recruited participants were made aware of the study's objectives, and their informed consent was obtained from each participant before enrollment. The participants did not receive any compensation for their time or participation.

### 2.2. Power Calculation

The minimum sample size to obtain reliable statistical information and draw inferences about the whole population was calculated as follows. An online formula for sample size estimation (Open Epi link using Kish) with a 95% significance level, 5% margin of error, and 50% response rate determined that the minimum representative sample size was 306 participants. Adjusting the sample size to account for potential drop-out considered as the nonresponse rate (attrition rate) was performed, and a 20% sample size was added. The response rate is the proportion of participants who responded to the survey, while the nonresponse rate is the proportion of participants who did not respond. The study adjusted for a 20% rate based on prior research and experience to account for potential drop-out. This helped ensure a reliable sample size for the study. In other words, the adjusted sample size equaled the estimated sample size/(1 − *W*), where *W* is the proportion expected to withdraw. Accordingly, 306/(1 − 0.2) resulted in 383 participants as the total sample size.

### 2.3. Selection Criteria for Recruiting Participants

The participants had to be undergraduate or postgraduate dental students registered in AU. There were no restrictions applied regarding the sex of the participant, nationality, or level of studies as long as they could understand English or Arabic. Students who were not registered with AU or could not read and understand English or Arabic language, as well as those from colleges other than dentistry, were excluded from this study.

### 2.4. Survey Instrument for Data Collection

A web-based self-administered structured questionnaire in English and Arabic was used to collect participant data. Anonymity was always preserved, and the trainees could stop their participation at any moment due to the voluntary nature of the survey and lack of incentive provided (i.e., no perceived benefit other than expressing their views about the subject matter). The questionnaire was prepared, distributed, and collected using an online survey software (SurveyMonkey, Momentive Inc., San Mateo, CA, USA). A web link collector generated the survey URL through which respondents could access the survey and send their answers. The questionnaire was composed of three sections. The first section provided information about the rationale and scope of the survey to permit data collection about vaccination, including candidate COVID-19 vaccines. Participants were reminded to respond with veracity, and each participant could reply to the survey once. The demographic data retrieved through the questionnaire was sex, age, place of residence, education level, and whether having a chronic condition (e.g., hypertension or diabetes).

The second section of the questionnaire assessed participants' COVID-19 knowledge and their willingness to be vaccinated (Would you be willing to be vaccinated when the COVID-19 vaccine is available? Yes, unsure, no), reasons for willingly being vaccinated against COVID-19, reasons for not wanting to be vaccinated against COVID-19, reasons for hesitancy to receive the COVID-19 vaccine, and factors which influence their acceptance, hesitancy, or rejection toward the vaccine to prevent COVID-19.

Cronbach's *α* coefficient for the questionnaire was calculated as 0.86 in a previous pilot study conducted with 20 trainees from the same institution. Participants who reported being likely to receive the vaccine were considered the vaccination-compliant group. In contrast, those who were unlikely to receive the vaccine were the noncompliant vaccination group. The third section of the questionnaire consisted of having the participants declare their sources of COVID-19 knowledge to have two research assistants (M.H.A. and E.M.E-A) qualify the reliability of the identified sources.

### 2.5. Statistical Analysis

The collected data were downloaded from online survey software as a spreadsheet (MS Excel 2010, Microsoft Corp., Redmond, WA, USA) and analyzed using statistical software (SPSS ver. 22; IBM Corp., Armonk, NY, USA). The present survey involved a close-ended questionnaire, which was validated using factor analysis. The internal reliability of all questionnaire tools was assessed by calculating Cronbach's *α*. An assumption of normality was established to check the validity of the parametric test using Shapiro–Wilk test. Significance in the Shapiro–Wilk test revealed *p* < 0.05; as a result, we rejected the null hypothesis, and the data were not normally distributed. Therefore, nonparametric tests were used to estimate the difference between the selected variables (gender and year of study) in relation to different parameters. The information was presented as frequency and percentage when assessing categorical variables. Descriptive statistics were applied to evaluate vaccine willingness according to the participants' demographic characteristics. The reasons for hesitating, desiring, or not desiring to be vaccinated were evaluated by sex. The chi-squared (*χ*^2^) test was used to evaluate the categorical variables. Nonparametric independent samples Mann–Whitney *U* test and Kruskal–Wallis test were used to determine significant differences in the distribution of all study parameters across study categories. The characteristics of the main reason for vaccine hesitancy and not being willing to be vaccinated were analyzed through logistic regression (LR) analysis (unstandardized regression coefficients (*β*), odds ratios (ORs), and their 95% confidence intervals (CIs)), with sex, age, and place of residence. The significance level was set at *α* = 0.05, with a 95% CI.

## 3. Results

### 3.1. Respondents' Characteristics

Out of the 882 invited students, 624 responded to the questionnaire (a response rate of 70.7%). Irrespectively, the participating sample population was almost two-fold larger than that required one. Most respondents were women (66.3%) and below 21 years of age (59.0%).

### 3.2. Knowledge of COVID-19 and Associated Determinants

Four hundred and twenty-four participants (72.4%) rated their COVID-19 knowledge level as “good” or “very good.” Most of the study participants (72.6%) were aware of the government (UAE) COVID-19 Task Force and mostly followed the UAE press conferences.

### 3.3. Determinants, Motivators, and Barriers to Vaccine Receipt among Dental Students

Although 60% of respondents would “probably” or “definitely” accept the vaccine immediately, a good number of respondents (34.2%) would “probably” or “definitely” avoid it. Among the top reasons given by the respondents to take the COVID-19 vaccine include the vaccine's effectiveness and minor side effects related to vaccine intake (Table 1). In contrast, two-thirds of the respondents indicated they would not take the vaccine until they knew more about how well it works (e.g., pharmacokinetics, pharmacodynamics, immunogenicity); likewise, one-third reported being concerned about possible side effects (e.g., safety, tolerability) related to vaccine use.

### 3.4. Association between the Level of Study and Vaccine Acceptance

First- and fourth-year students have been the least hesitant to vaccinate; most (67.0% and 73.0%) indicated they would receive vaccination immediately (Table 2). Over half of 3rd- and 5th-year students reported being willing to receive the vaccine immediately if it were available. Interestingly, more than half of the 2nd-year students would reject the vaccination if they had an option with a significant difference between the independent groups when using the Kruskal–Wallis *H* test (*p* < 0.001) (however, it is mandatory unless a medical exception is granted from the Ministry of Health and Prevention).

### 3.5. Association between Student Sex and Vaccine Acceptance

Despite efforts to specifically reduce vaccine fears among students, only 6 out of 10 women and men would opt to be vaccinated immediately. Mann–Whitney test reveals no significant difference (*p* = 0.849) when comparing male to female students in relation to their level of compliance to the COVID-19 vaccine (60.9% and 59.4%, respectively) (Table 2).

### 3.6. Association between Student Age and Vaccine Acceptance

Mature students remained the most eager to become vaccinated. The older students were significantly (*p* ≤ 0.001) more likely to support their immediate vaccination (Table 2). However, the sample of the older group was significantly smaller than the younger groups. Still, 6 out of 10 trainees under 24 would be vaccinated immediately (Table 2). More detailed data in relation to dental trainees' level of knowledge and attitude was mentioned in Table 2.

**Figure 1:**
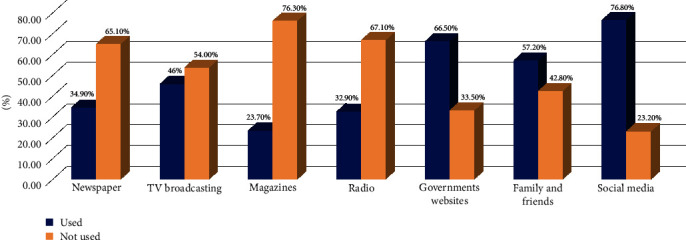
Sources of information about COVID-19 (*what are your sources of information for COVID-19?*).

LR analysis was chosen to ascertain the relationship between attitude, knowledge, and compliance in relation to different parameters. In addition to the previous analysis, more codes were given to attitude, knowledge, and compliance as positive and negative concerning the previous criteria of the scoring methods. Multiple binary LR analysis revealed that older age (OR: 1.7, 95% CI: 0.621–4.671) was a positive but not significant predictor of the probability of vaccine compliance with the OR, indicating that for every unit increase in the predictors, the OR increases by 1.703 (Table 2). The reported top three sources of COVID-19-related information were social media (76.8%), government websites (66.5%), and family and friends (57.2%), as displayed in Figure 1.

## 4. Discussion

The satisfactory response rate obtained in this study shows the extraordinary reach of administering questionnaires. Compared with previous similar studies [18, 19], the present findings were more favorable regarding the incidence and circumstances surrounding COVID-19. In this study, most students (72%) had adequate knowledge of COVID-19. This finding is comparable to reports from other countries where students showed excellent knowledge of COVID-19 virus infection [18]. However, 6 out of 10 participants projected a positive attitude toward the COVID-19 vaccine. Furthermore, it can be inferred that dental surgery trainees may lack proper knowledge in some respects of COVID-19 despite the amount of information available. Thus, this concern must be prioritized by academic institutions and the health authorities so that students are educated with the needed resources and to stimulate their awareness of the pandemic.

This web-based survey study provides similar results to other research [20–24], as vaccine effectiveness was linked to the likelihood of accepting becoming vaccinated. Studies about coronaviruses show that lifetime immunity is improbable [21, 22, 25]. Our study is also alighted with other research demonstrating how influential the opinions can be of people's vaccination willingness, especially from friends and relatives, local medical doctors, and public health authorities [20, 26, 27]. Similar to previous studies on influenza vaccine acceptance [28, 29], younger and female respondents were significantly less willing to become vaccinated against COVID-19 (despite the difference with the male was only 0.5% less). In contrast, educational attainment was linked with vaccination acceptance. Correspondingly, public health establishments could contemplate approaches that tackle the particular interests of female and younger students more susceptible to being skeptical about the vaccine. The great number of students in our sample that support the COVID-19 vaccine is a surprising finding since, previously, patients expressed great concern for the vaccines' safety [30, 31], which demands an extended assessment period. The apparent risk of vaccines has been correlated with an unwillingness to embrace vaccination [32]. It is a fact that numerous barriers are associated with vaccine hesitancy [33], including trust in healthcare providers' advice, social network influences, knowledge about vaccines, and general views toward health [34].

This study was conducted early in 2022, with daily media rumors of the death stats and speedy breakthroughs concerning the disease and its variants. The distress of the pandemic and its overwhelming effects may have altered dental medicine students' agreement to less strict by-laws. Comparably, panic concerning the swine flu was related to amplified H1N1 vaccine acceptance [35, 36]. Experimental vaccine formulation used as an emergency and an alteration in risk–benefit due to elevated illness and death have been proposed as adequate [37]. Though some respondents are disturbed about the side effects of vaccination, possibly beyond the disease itself [38], more side effects during a pandemic could be adequate from a public health perception [37]. A critical factor that may impact trainees' motivation was the belief that the vaccine is effective, that many people experienced only minor side effects, and that the disease is essentially a self-limiting, benign disease [39]. Therefore, understanding trainees' attitudes toward COVID-19 vaccination assessment and authorization process should be prioritized. This data may aid in updating public health announcements and plans for cultivating society's approval when a more accessible COVID-19 vaccine is available.

The main sources of COVID-19-related knowledge were social media and official government websites. This means the online COVID-19-related updates released by the official health advisory board have significantly improved dental surgery trainees' awareness. This is encouraging because using authentic sources for COVID-19 information and notifications is crucial in providing students with transparent information and preparedness. Moreover, many students use social media to stay updated concerning COVID-19. This might be of notable concern as many authorities have highlighted the quality of social media information [40–42]. Similarly, different centers have reported that social media is the major source of information, followed by television [43–45]. Despite social media being a cost-effective and easily accessible source of information, it might spread false information and affect the audience's beliefs and decisions [46]. Therefore, healthcare authorities must ensure the availability and approachability of authentic information to the public, including students. Furthermore, the clinical trainees should be familiar with and carefully evaluate COVID-19-related websites and any awareness materials before sharing or implementing them to avoid misinformation or malpractice [47, 48].

The WHO declared vaccine hesitancy as a major threat to global health. Vaccination unwillingness considerably originated from erroneous ideas of contagious diseases and vaccines as different coronavirus strains/variants emerge. Likewise, the International Association of Dental Students [49] conducted a machine-learning analysis using data derived from 22 countries and recommended some predictors of COVID-19 vaccination desirability as follows:

- the financial status of the state where the student lives,
- the trust in the pharmacological business,
- the misunderstanding of natural immunity,
- the trust in vaccines' risk–benefit ratio, and
- the acceptance likelihood of new vaccines.

Studies have shown that students, like other populations, have varying levels of acceptance of the COVID-19 vaccine. The study by Karbasi et al. [50] surveyed undergraduate students in the United States and found that around 60% of students were willing to vaccinate against COVID-19. The study also found that younger students were more unlikely to be willing to vaccinate compared to older students. The authors concluded that age is an important factor in the acceptance of the vaccine by students. Another study by Patel et al. [51] surveyed medical students in India and found that around 80% of students were willing to vaccinate against COVID-19. The authors found that the students' willingness to vaccinate was influenced by their perceptions of the safety and effectiveness of the vaccine, as well as their trust in the medical establishment. The authors concluded that education and awareness campaigns aimed at addressing these concerns could increase the acceptance of the vaccine among students.

A study by Chan et al. [52] surveyed nursing students in Hong Kong and found that around 70% of students were willing to vaccinate against COVID-19. The authors found that the students' willingness to vaccinate was influenced by their perceptions of the safety and effectiveness of the vaccine, as well as their trust in the medical establishment. The authors concluded that education and awareness campaigns to address these concerns could increase students' acceptance of the vaccine.

In addition to age and perceptions of safety and efficacy, other factors such as personal beliefs, cultural values, and religious beliefs can also influence the acceptance of the vaccine by students. A study by Singh et al. [53] surveyed college students in India and found that religious beliefs were an important factor in the acceptance of the vaccine. The authors found that students who held strong religious beliefs were less likely to be willing to vaccinate compared to those who did not.

Furthermore, Kateeb et al. [54] assessed the predictors related to the willingness of dental surgery trainees to receive the COVID-19 vaccine in another study and established that suitable reports about vaccines, their risk–benefit, and immunity (e.g., natural and acquired) are essential to increase trust and foster better mindsets about vaccines in the future generations of dental clinicians. Additionally, the vaccine hesitancy reasons among Czech undergraduate trainees suggested a decent likelihood of reaching herd immunity [55]. It was concluded that the primary prevention measures in the Czech Republic should be culturally profound and inclusive. To sum up, it is the target recommended that students stay vigilant against new, more transmissible virus strains and continue to adhere to preventive measures to reduce transmission of the disease despite the continued increase of vaccine distribution and production.

The problem addressed by this study is the low COVID-19 vaccination uptake among the student sector of healthcare professions, including dental medicine trainees (<75% vaccinated). Understanding the factors influencing vaccine acceptance among dental students may inform public health authorities about the types of endorsements, incentives, or messages necessary to increase community uptake.

### 4.1. Limitations

There are limitations inherent to the cross-sectional design that are difficult to avoid completely. First, the data presented depend on the trainees' integrity and recall ability, so there is a high risk of misrepresenting information as they rely on their memories to answer the survey. Second, the students shared their anxieties during a major change in daily activities (virtual teaching/learning) [56–61], and their insights of a shortened vaccine progress may differ when the college teaching and clinical training returns to normal and the number of infected patients drops. Given the unique stressors during this period when the understanding of this disease was evolving and the remarkable fear of harm, these findings may have overestimated the trainees' actual approval of an accelerated COVID-19 vaccine [62, 63]. The older age group, significantly more associated with being vaccine-ready than the younger ones, contained less than 5% of the total respondents. Although the women doubled the men participants, the sex proportion of students enrolled in dental medicine is the same. Given these limitations, further longitudinal studies should be warranted to examine the understanding, attitude, and practice of COVID-19 in different countries.

Findings in the current study may help inform health promotion to dissipate vaccine hesitancy. In addition, academic institutions are responsible for facilitating successful student vaccination programs to protect patients, allied health professionals, and staff. Further multinational longitudinal studies should identify the dental medicine trainees' reasons and willingness to receive the COVID-19 vaccination.

## 5. Conclusion

Within the limitations of the cross-sectional study, the findings indicated that:

- Dental surgery trainees in Ajman have moderate knowledge of COVID-19 infection.
- Students' primary sources of COVID-19-related information were social media, government websites, and family and friends.
- Age (24 years and up), sex (male), and study year (1st and 4th) had a more positive response when asked about the acceptance to receive the vaccination.
- The most important reason for refusing the vaccine is not knowing how it works, followed by the fear of side effects and complications related to the vaccine.
- Various factors, including age, perceptions of safety and efficacy, and personal beliefs, influence the acceptance of the COVID-19 vaccine by students. Education and awareness campaigns aimed at addressing these concerns can increase the acceptance of the vaccine among students.

## Figures and Tables

**Table 1:** Reasons for accepting or refusing to take the vaccine.

Willingness	Acceptance/reasons	Frequency (%) ^*∗*^
*If a vaccine to prevent COVID-19 were available today*, *you would:*	Definitely vaccinate	144 (23.1%)
	Probably vaccinate	230 (36.9%)
	Probably NOT vaccinate	170 (27.2%)
	Definitely NOT vaccinate	50 (8%)
	No answer	30 (4.8%)

*Refuse the vaccine*	Concern about side effects	212 (34.0%)
	Do not think I need it	66 (10.6%)
	It would cost too much	66 (10.6%)
	Want to know more about how it works	420 (67.3%)

*Accept the vaccine*	You had to pay out-of-pocket to obtain it	227 (36.4%)
	The vaccine was effective about 60% of the time	304 (48.7%)
	Many people experienced minor side effects	262 (42.0%)
	You needed to vaccinate again every year or so	196 (31.4%)

^*∗*^Participants could select more than one reason.

**Table 2:** Vaccine compliance by dental surgery trainees.

	Positive response, *n* (%) ^*∗∗*^	Negative response, *n* (%) ^*∗∗*^	*p*-value^*∗*^	Logistic regression	
				Odds ratio (CI)	Significance of OR^*∗∗*^
Knowledge					
Gender					
Male	134 (63.8%)	76 (36.2%)	0.069	1	0.115
Female	290 (70.0%)	124 (30.0%)		1.326 (0.933–1.885)	
Age range					
18–20 years old	244 (66.3%)	124 (33.7%)	0.071	1	
21–23 years old	154 (68.1%)	72 (31.9%)		1.045 (0.489–2.234)	0.909
24–26 years old	26 (86.7%)	4 (13.3%)		2.356 (0.687–8.157)	0.172
Year of study					
First year	122 (64.9%)	66 (35.1%)	0.001	1	
Second year	88 (75.9%)	28 (24.1%)		1.660 (0.985–2.796)	0.057
Third year	30 (50.0%)	30 (50.0%)		0.539 (0.299–0.974)	0.230
Fourth year	98 (77.8%)	28 (22.2%)		1.627 (0.735–3.598)	0.040
Fifth year	86 (64.2%)	48 (35.8%)		0.910 (0.384–2.157)	0.830

Attitude					
Gender					
Male	190 (51.3%)	180 (48.7%)	0.577	1	0.577
Female	378 (52.5%)	342 (47.5%)		1.014 (0.553–1.862)	
Age range					
18–20 years old	328 (51.3%)	310 (48.7%)	0.168	1	
21–23 years old	214 (53.2%)	188 (46.8%)		1.883 (0.536–6.614)	0.323
24–26 years old	26 (52%)	24 (48%)		1.579 (0.660–3.777)	0.305
Year of study					
First year	172 (53.1%)	152 (46.9%)	0.061	1	
Second year	104 (52%)	96 (48%)		0.815 (0.364–1.826)	0.619
Third year	48 (47.1%)	54 (52.9%)		0.357 (0.157–0.808)	0.081
Fourth year	118 (52.7%)	106 (47.3%)		0.760 (0.356–1.624)	0.963
Fifth year	126 (52.5%)	114 (47.5%)		0.838 (0.193–3.638)	0.713

Vaccine compliance					
Gender					
Male	128 (60.9%)	82 (39.1%)	0.849	1	0.324
Female	246 (59.4%)	168 (40.6%)		0.833 (0.580–1.197)	
Age range					
18–20 years old	218 (59.2%)	150 (40.8%)	≤0.001	1	
21–23 years old	134 (59.3%)	92 (40.7%)		1.136 (0.551–2.342)	0.729
24–26 years old	22 (73.3%)	8 (26.7%)		1.703 (0.621–4.671)	0.301
Year of study					
First year	126 (67%)	62 (33%)	≤0.001	1	
Second year	52 (44.8%)	64 (55.2%)		0.375 (0.230–0.610)	0.065
Third year	36 (60%)	24 (40%)		0.727 (0.398–1.328)	0.300
Fourth year	92 (73%)	34 (27%)		1.127 (0.530–2.397)	0.756
Fifth year	68 (50.7%)	66 (49.3%)		0.425 (0.186–0.974)	0.043

^*∗*^*p*-value, Mann–Whitney and Kruskal–Wallis test;  ^*∗∗*^*n* (%), frequency (%); CI, confidence interval.

## Data Availability

Raw data are available upon request.
